# Perinatal transmission of dengue: a case report

**DOI:** 10.1186/1756-0500-7-795

**Published:** 2014-11-14

**Authors:** Vindika Prasad Sinhabahu, Rajeev Sathananthan, Gathsaurie Neelika Malavige

**Affiliations:** Special Care Baby Unit, Colombo South Teaching Hospital, Kalubowila, Sri Lanka; Centre for Dengue Research, Faculty of Medical Sciences, University of Sri Jayawardanapura, Nugegoda, Sri Lanka; Department of Microbiology, Faculty of Medical Sciences, University of Sri Jayawardanapura, Nugegoda, Sri Lanka

**Keywords:** Maternal dengue, Perinatal dengue, Vertical transmission

## Abstract

**Background:**

Dengue in pregnancy is associated with many maternal and foetal outcomes including perinatal transmission of dengue infection.

**Case presentation:**

A baby was born by emergency caesarean section due to foetal distress and meconium stained liquor, to a 27-year old primi-gravidae, Sinhalese female, who was febrile during and 2 days prior to labour. The baby had evidence of respiratory distress due to meconium aspiration and was cared for in the special care baby unit for 3 days. On the 4^th^ day he developed fever and serial blood counts showed a gradual rise in the haematocrit (>20% of baseline value) and lowering of platelet counts. The baby was treated for sepsis and as Sri Lanka was experiencing a massive dengue epidemic was also tested for dengue. His dengue NS1 antigen test was strongly positive and the dengue IgM antibodies weakly positive on day 3 of illness. The mother was positive for both dengue IgM and IgG antibodies.

**Conclusion:**

Although rare, vertical transmission of the dengue virus has been reported and the baby most likely developed dengue due to perinatal transmission of dengue.

## Background

Dengue viral infections are estimated to infect 390 million annually resulting in significant morbidity and mortality in resource poor countries [1]. Infection can occur due to any of the four dengue viruses which may manifest as asymptomatic infection, undifferentiated fever, dengue fever or result in severe clinical disease manifestations in the form of dengue haemorrhagic fever (DHF)/ dengue shock syndrome (DSS) or dengue infection complicated with organ failure [2]. Although majority of individuals infected with the dengue virus develop asymptomatic disease, infection during pregnancy has shown to be associated with more severe disease and higher incidence of death [3]. Dengue in pregnancy is associated with preterm delivery, intra-uterine death, miscarriages and acute foetal distress during labour [4]. Apart of foetus adversely been affected by complications due to maternal dengue, the foetus can directly be infected with the virus due to perinatal transmission.

Dengue in the newborn has shown to cause a range of clinical symptoms from asymptomatic infection, mild disease, DHF or DSS [5]. We report a baby who was born by an emergency caesarean section due to foetal distress developing dengue due to perinatal transmission of the virus.

## Case presentation

A baby was born by emergency caesarean section due to foetal distress and meconium stained liquor, to a 27- year old primi gravid, Sinhalese female. The mother had fever during delivery and had been febrile 2 days prior to delivery. The baby had respiratory distress at birth and his umbilical cord was stained with meconium. The chest radiograph revealed evidence of meconium aspiration. The baby was given incubator care and was started on intravenous crystalline penicillin and gentamycin. Initially as the baby had features of meconium aspiration syndrome, he was kept off any fluids by mouth and was given intravenous 10% dextrose 60 ml/kg/day with Calcium Gluconate. The baby’s respiratory distress gradually resolved and he weaned off and sent to cot care on day 3. At this point the baby was commenced on expressed breast milk every 3 hours. He remained afebrile in first 4 days. On day 5 of life, he developed fever spikes (102°F). The baby’s C-reactive protein was normal. Serial full blood counts showed gradual decline in platelet counts and a >20% rise in the PCV. At this stage, total intravenous fluids were gradually increased to 150 ml /kg and continued at a rate of 150 ml/kg/day. The baby’s pulse rates, capillary refilling time, warmth of peripheries, urine output were closely monitored to detect any haemodymanic instablity. Breast feeding was started on day 5 of illness and was fully established by day 8 of illness. IV fluids were gradually reduced and omitted on day 8 of illness. The baby’s serial full blood counts are shown in Table 1. As sepsis or a viral infection was suspected the baby was given cefotaxime intravenously.

**Table 1:** **Results of serial haematological parameters and other laboratory investigations of the patient**

Date	At birth	D1	D2 morning	D2 evening	D3	D4	D5	D7	D8	D11
WBC	17300	3930	16870	21790	15300	12360	10180	14030	12280	12630
N%	82%	74%	60	68	46	36	36	38	24	23
L%	14%	16	38	27	43	51	52	49	64	64
Platelets	202	38	23	18	25	16	16	30	31	212
Hb	14.9	14.8	15.4	14.7	15.6	25.2	20.3	14	14.5	12.3
PCV	42.7	43.2	41.9	42.8	44.5	75.5	65.2	40.7	45	38

D1 indicates day 1 of illness which is the 4^th^ day of life.

As the mother was febrile during delivery, possible perinatal dengue was suspected. The baby’s dengue NS1 antigen test was strongly positive on day 3 of fever and the dengue specific IgM antibodies were weakly positive. The PCR was negative. The mother was also tested at the same time and both dengue IgM and IgG were positive. The baby’s blood culture did not yield a bacterial growth and the blood picture showed evidence of viral infection. The liver enzymes were elevated. He was managed as having dengue haemorrhagic fever according to the 2011 WHO guidelines [6]. During the clinical course, he did not develop any evidence of haemorrhage or pleural effusions or ascites. He was discharged on Day 14 of life.

## Discussion

Fever in a newborn especially following intensive care due to meconium aspiration is usually considered to be due to sepsis. However, especially in dengue endemic countries maternal dengue in common [3] but could be potentially not diagnosed as an acute dengue infection, especially if the clinical symptoms are that of a non specific febrile illness. Sri Lanka has been affected by epidemics of dengue infection for almost three decades and many maternal dengue infections were initially misdiagnosed as complications due to pregnancy and even pulmonary embolism [7, 8]. Apart from miscarriages in 2 women, perinatal dengue was not reported in both of these case series.

Perinatal transmission of dengue although rare, has been reported previously [4, 5, 9, 10]. Perinatal dengue was thought to be transmitted via the placenta as the virus has been isolated in placentas and in cord blood of such infants [5]. However, recently it was also shown that the dengue virus may be transmitted by breast milk, as the virus was detected in breast milk but not in cord blood [11]. Once the dengue virus infects a human, the incubation period is thought to be between 3–10 days [12]. Since this baby developed dengue infections four days following delivery, he is most likely to have been infected perinatally. Although the mother did not have a severe clinical disease, the dengue infection could have resulted in foetal distress leading to meconium aspiration. Virus isolation or dengue antibody detection was not carried out on cord blood or placenta in this instance, as dengue infection was not suspected at the time of delivery.

## Conclusion

In conclusion, although perinatal dengue is a rare entity, it should be suspected in neonates whose mothers were suffering from a febrile illness during or just before delivery. Since perinatal dengue has been shown to associate with foetal distress, preterm delivery and intra uterine death, early identification of this condition is crucial to prevent development of complications.

## Consent

Written informed consent was obtained from the patient’s father for publication of this Case Report and any accompanying images. A copy of the written consent is available for review by the Editor-in-Chief of this journal.
